# Deep Learning Approach for Automatic Heartbeat Classification

**DOI:** 10.3390/s25051400

**Published:** 2025-02-25

**Authors:** Roger de T. Guerra, Cristina K. Yamaguchi, Stefano F. Stefenon, Leandro dos S. Coelho, Viviana C. Mariani

**Affiliations:** 1 Graduate Program in Electrical Engineering, Federal University of Parana, Curitiba 80242-980, PR, Brazil; stefano@uniplaclages.edu.br (S.F.S.); leandro.coelho@ufpr.br (L.d.S.C.); 2 Postgraduate Program in Productive Systems in Association with UNIPLAC, UNC, UNESC, and UNIVILLE, Lages 88509-900, SC, Brazil; cristinayamaguchi@uniplaclages.edu.br; 3 Department of Electrical Engineering, Federal University of Parana, Curitiba 80242-980, PR, Brazil; viviana.mariani@ufpr.br; 4 Graduate Program in Mechanical Engineering, Federal University of Parana, Curitiba 80242-980, PR, Brazil

**Keywords:** cardiac arrhythmia detection, multiclass classification, deep learning

## Abstract

Arrhythmia is an irregularity in the rhythm of the heartbeat, and it is the primary method for detecting cardiac abnormalities. The electrocardiogram (ECG) identifies arrhythmias and is one of the methods used to diagnose cardiac issues. Traditional arrhythmia detection methods are time-consuming, error-prone, and often subjective, making it difficult for doctors to discern between distinct patterns of arrhythmia. To understand ECG signals, this study presents a multi-class classifier and an autoencoder with long short-term memory (LSTM) network layers for extracting signal properties on a dataset from the Massachusetts Institute of Technology and Boston’s Beth Israel Hospital (MIT-BIH). The suggested model had an accuracy rate of 98.57% on the arrhythmia dataset and 97.59% on the supraventricular dataset. In contrast to other deep learning models, the proposed model eliminates the problem of the gradient disappearing in classification tasks.

## 1. Introduction

Heart diseases, also known as cardiopathies, indicate alteration in the heart of pathological origin, capable of causing abnormal heart rhythms, or arrhythmias, which can be detected through disturbances in its electrical activity. Arrhythmias are classified into tachyarrhythmias and bradyarrhythmias. In the former, the heart rate can exceed 100 beats per minute, while in bradyarrhythmias, the rate is below 60 beats per minute. However, an arrhythmia can occur in isolation, so the heart rate can be regular during the event. Second [1], many arrhythmias are caused by premature beats, also known as ectopic beats, which show morphological changes in electrocardiographic recording. This beat is caused by abnormal electrical activation and can originate in the region of the atria, at the atrioventricular junction, or in the region of the ventricles.

Cardiac arrhythmias can cause morbidity and mortality. For example, as the most common arrhythmia, atrial fibrillation increases the risk of stroke [2]. Several mechanisms appear to contribute to the concomitant presence of atrial fibrillation, including advanced age. These heart diseases affect more than four million people in the United States of America, generating a health cost that becomes a substantial economic burden [3]. The population’s age worldwide is predicted to rise, and by 2030, the number of adults over the age of 60 is predicted to rise by 56%. In addition, the growing population of 60 and older has been estimated at 2.1 billion by 2050.

This increase in the elderly population has presented itself as an economic and health challenge for the world [4], since the human cardiovascular system becomes prone to disease and weakens as a person ages, as well as the arteries and muscular wall of the left ventricle thickening and shrinking with age, resulting in a decrease in the compliance of blood vessels in the arteries.

Due to differences in electrocardiogram (ECG) patterns due to age, gender, ethnicity, and underlying medical disorders, existing ECG classification models generally have problems generalizing across different groups. When models trained on one dataset perform significantly worse when applied to another, this limitation is particularly evident [5]. Class imbalance is an observed characteristic of ECG datasets, where specific diagnostic categories are considerably underrepresented in comparison to others. This imbalance is an important issue since it sometimes produces biased models that perform well for majority classes but worse for minority classes in terms of generalization and diagnostic accuracy [6].

Patient movement, improper electrode placement, or equipment interference may all add noise and irregularities into ECG data. Misclassification results from existing approaches’ inability to handle these disturbances robustly [7]. Without understanding the logic behind these models’ predictions, clinicians may be hesitant to accept them [8].

Because annotation is costly and requires specialized expertise, outstanding labeled ECG datasets tend to be small. This affects the way that deep learning models perform, as they require a lot of labeled data to operate most effectively [9]. Many ECG classification methods are highly computational and might not be appropriate for applications that operate in real time, including continuous monitoring systems or wearable technology [10].

Diseases caused by disturbances in the heartbeat pattern can be identified by an ECG, so it is an essential step for tasks in clinical practice because the ECG is a non-invasive test frequently performed to assess the condition of the heart. Furthermore, ECG data are useful in the identification of cardiovascular illnesses such as atrial fibrillation, myocardial infarction, congestive heart failure, and premature contractions of the atria or ventricles [11]. The amount of ECG data that need to be analyzed has grown rapidly and can be screened by human cardiologists.

ECG classification is typically handled by a Convolutional Neural Network (CNN) using wavelet transform to improve feature extraction. By increasing the number of layers, the CNN’s learning capacity gradually increases. Increasing the number of layers does not improve the accuracy of the model. Gradient fading in CNNs is increasingly exposed as the number of layers increases. Several approaches have been offered to overcome this issue, such as normalized initialization or intermediate normalized methods [12]. However, the model’s accuracy is decreased due to increased model layers. It is worth mentioning that long short-term memory (LSTM) network does not present this issue and seems to outperform CNNs in most cases.

We consider the challenges in understanding the existing deep learning (DL) models and the difficulties when evaluating noisy, unbalanced data. Based on recurrent networks, this research proposes an effective model for ECG classification that enhances classification performance due to its more compact architecture.

To extract signal properties from the Massachusetts Institute of Technology and Boston’s Beth Israel Hospital (MIT-BIH) dataset; reduce noise; identify signals; and classify the detection of anomalies present in heartbeats, such as premature atrial or ventricular contractions, left or right bundle branch blocks, and normal beats, this article aims to present a multi-class classifier and an autoencoder with LSTM network layers.

This is how the rest of the paper is structured: In Section 2, a literature review focused on techniques for detection of arrhythmias is presented. In Section 3, the problem description that gives the motivation of the application covered here and the considered dataset are described. In Section 4, the algorithm, which consists of a filter to treat noise combined with an autoencoder built by an LSTM network and the classifier, is explained, and the setup for comparison is detailed. In Section 5, the main results are presented. In Section 6, a discussion is presented. Finally, in Section 7, the conclusion and future work directions are presented.

## 2. Literature Review

The main objective of this section is to look for studies carried out between 2010 and 2024 on the classification of cardiac arrhythmias. Then, the inclusion and exclusion criteria are defined, as are the strategies for handling the search in the dataset, the critical evaluation of the papers, and the selection of studies to be included in the review. The specified requirements for inclusion were (i) having disease detection in scope, (ii) being focused on the classification of cardiac arrhythmias, and (iii) using the MIT-BIH dataset. The exclusion criteria defined were articles that (i) are not in the English language, (ii) are not on ECG data, and (iii) do not use machine learning (ML) or DL models.

During the eligibility phase, following the aspects mentioned previously, it was possible to reduce the total to 199 articles, of which 84 (43%) were published before 2019, 112 (58%) after 2019, 116 in the area of biomedical engineering, and 25 (13%) in the area of artificial intelligence. Fifty-two articles were selected that met the inclusion criteria and addressed the detection of heart disease and arrhythmias using the MIT-BIH dataset [13].

**Figure 1:**
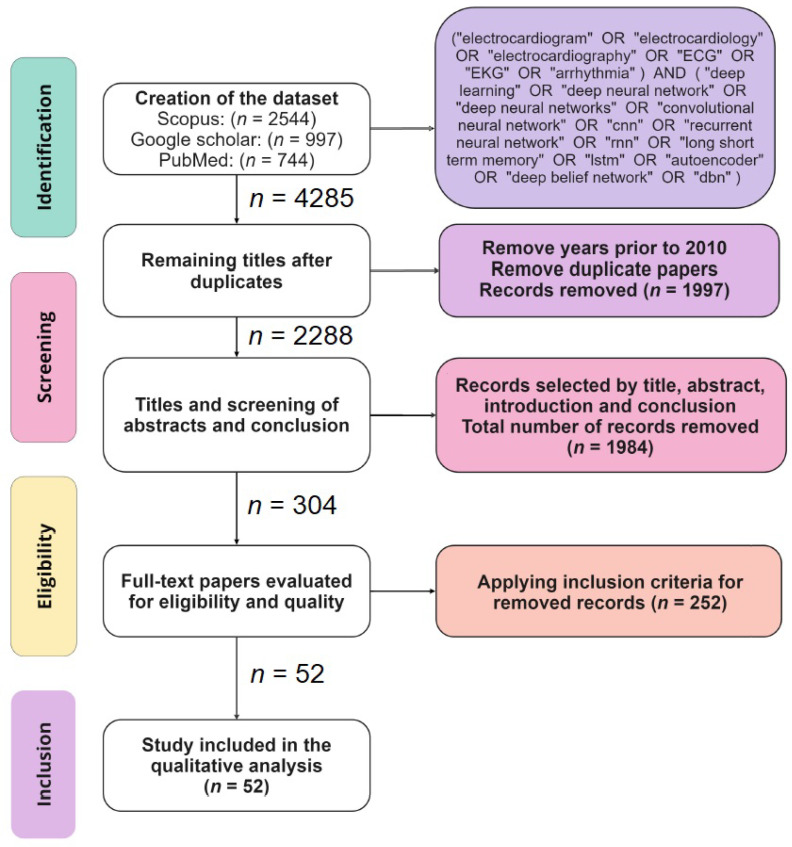
Diagram for identifying and adding papers to the literature review of this study.

These articles were selected because they demonstrated the main approaches used in the automatic classification of cardiac arrhythmias, in addition to using a common dataset and presenting a comparative study with other models found in the literature. After 2022, it was possible to identify a lack of publications, as they presented a dataset different from the one chosen as validation for this study. To address a comprehensive literature review, in this study, a search process was considered, as illustrated in Figure 1. This process consisted of four stages related to identification, screening, eligibility, and inclusion of the review.

One of the most significant uses of artificial intelligence (AI) in ECG analysis continues to be the detection of arrhythmias. The complexity and variety of ECG data are sometimes too much for conventional strategies to process, but AI models—in particular, CNNs and recurrent neural networks (RNNs)—have demonstrated outstanding performance. For instance, Attia et al. [14] demonstrated the use of CNNs to identify atrial fibrillation from single-lead ECG data with high accuracy, outperforming conventional algorithms. More recently, Ribeiro et al. [15] proposed a deep learning model that achieved state-of-the-art results in detecting multiple arrhythmias using a large-scale ECG dataset. These studies underscore the potential of AI in improving diagnostic accuracy and reducing manual intervention.

ECG signal quality is necessary for reliable diagnosis, and AI is being used more and more for assessing and enhancing signal quality. Recent work by Clifford et al. [16] introduced a DL-based framework for automated signal quality assessment, which effectively distinguishes between clean and noisy ECG recordings. This approach has been integrated into wearable devices, enabling real-time quality monitoring and improving the reliability of remote ECG monitoring systems.

In ECG analysis, AI has also been utilized for predictive analytics, particularly in the prediction of cardiovascular events. Tison et al. [17] developed a DL model capable of predicting the risk of heart failure and other cardiac conditions using raw ECG data. Their model demonstrated superior predictive performance compared to traditional risk scores, highlighting the potential of AI in preventive cardiology. Similarly, Kwon et al. [18] proposed a transformer-based model for predicting myocardial infarction from ECG signals, showcasing the adaptability of advanced AI architectures in this domain.

Matias et al. [19] presented a review of studies using AI for atrial fibrillation prediction, referencing studies from 2009 to 2019, containing ML and DL models. In addition, more than half of the selected studies were published after 2016, corroborating that the topic of this study is recent and has potential for future research.

Parvaneh et al. [20] review studies from 2011 to 2019, which present DL models in the detection of arrhythmias. Ebrahimi et al. [21] applied DL models to the ECG signal for classification purposes, considering studies from 2017 to 2018; they observed that the CNN model is predominantly appropriate for feature extraction, which was noted in 52% of the studies. Hong et al. [22] reviewed studies from 2010 to 2020, focusing on DL models applied in various ECG analyses, detection, disease classification, annotation, localization, sleep staging, human biometric identification, and noise elimination. They noted that hybrid architectures containing a CNN and RNN allowed for more reliable results.

Due to the ability to automatically extract features, several DL models are used, such as the CNN, with several convolutional layers, followed by the batch normalization, activation function, output, pool, and classification layers, as described in Hannun et al. [23].

Xia et al. [24] addressed CNNs applied for automatic atrial fibrillation detection; however, the two-dimensional parameter input structure was essential for deep networks. The ECG is a one-dimensional time-varying signal, which does not meet the input structure requirement. To solve these problems, stationary wavelet transform was adopted for ECG preprocessing. The processed signal is reorganized into a two-dimensional parameter structure to meet the input structure requirement of deep networks.

Pourbabaee et al. [25] studied a large volume of ECG time series used as input to the CNN, which learned the representative and main characteristics of paroxysmal atrial fibrillation and simplified the extraction process of characteristics corresponding to different cardiac arrhythmias. Zhai and Tin [26] and Xia et al. [24] transformed heartbeats into numerical matrices used as inputs to the CNN classifier. They captured the morphology of the beats and the correlations in the ECG. They also selected more representative beats for CNN training, improving classification performance.

Residual Neural Networks (ResNet), Dense Convolutional Networks (DenseNet), and Inception models are pre-trained on large, domain-specific datasets, leveraging their distinct architectural designs to maximize performance and improve their capacity to extract highly discriminative and meaningful features from complex data. ResNet utilizes skip connections to mitigate vanishing gradients and enable deeper architectures, DenseNet enhances feature reuse through dense layer-wise connections, and Inception models employ multi-scale convolutional filters to capture diverse patterns efficiently. These models can be fitted to an ECG dataset to find heartbeat disturbances, as discussed by Andreotti et al. [27]. This segment represents the onset of ventricular repolarization corresponding to the slow repolarization phase in a plateau of ventricular myocytes, representing a period of inactivity between depolarization and the onset of ventricular repolarization. Alterations in this segment are of importance in diagnosing acute coronary syndromes, also known as arrhythmias.

In addition to CNNs, there are applications in the area using recurrent neural networks (RNNs) that are primarily designed for use on sequential data, time series, event sequences, and natural language processing Wołk and Wołk [28]. Particularly, for ECG data, RNNs can be used to capture time-dependent data and process inputs with different lengths.

Maknickas and Maknickas [29] considered an LSTM network that learns patterns directly from precomputed features to classify between normal and atrial fibrillations. Chang et al. [30] explored the high-order spectral and temporal characteristics of multi-lead ECG signals from patients with atrial fibrillation by applying an LSTM network, demonstrating that the deviation of temporal variations is critical for the detection of atrial fibrillation. Schwab et al. [31] applied RNNs to identify temporal and morphological patterns in segmented ECG recordings. In Zhou et al. [32], the LSTM network was explored for a premature ventricular contraction detection problem.

Rajan and Thiagarajan [33] developed a generative modeling approach for ECG classification, allowing the use of unsupervised data and also providing highly robust metric spaces suitable for subsequent discriminative learning. Liu and Kim [34] used an LSTM network and explored the use of a symbolic aggregation approach in the data preprocessing stage to improve accuracy. Yildirim [35] presented a new model for wavelet sequences generated in a deep bidirectional LSTM network to classify ECG signals.

Saadatnejad et al. [36] used two LSTM networks to combine raw ECG signals and wavelet transform the features. Li et al. [37] incorporated an attention mechanism into a bidirectional LSTM (Bi-LSTM) network for handling long-duration ECG signals with varying sequence lengths and multichannel inputs. Convolutional RNN, a fusion between CNN and RNN, was proposed by Hong et al. [38] such that the CNN is used to extract features from an ECG signal and the RNN is used to summarize the features over the time dimension, generating global features. The stacked denoising autoencoder [39], stacked autoencoder [40,41], and convolutional autoencoder [42] have been used for ECG denoising purposes, as noises like baseline drift, electrode contact noise, and motion artifacts can skew ECG readings and produce incorrect interpretations.

Farhadi et al. [43] presented a combination of deep learning models that aims to classify atrial fibrillation using an ECG signal by extracting spectral, temporal, and nonlinear features from the signal; it was also used in parallel to classify atrial fibrillation and normal samples. Wang et al. [44] presented a stacked denoising autoencoder as in Xia et al. [39] but used a Bi-LSTM classifier to take full advantage of the temporal information of the data, achieving heartbeat classification. Yildirim et al. [42] presented a compression framework to reduce the size of the heartbeat signal by employing LSTM to automatically recognize arrhythmias using ECG features.

During the literature review, it was possible to identify studies using hybrid architectures, for example, applying CNN with RNN, some articles using autoencoder, and using CNN together with other techniques in noise reduction and feature extraction problems. It is worth mentioning that no articles were obtained that used autoencoder approaches with LSTM layers.

## 3. Dataset

The MIT-BIH dataset includes 47 people’s 48 half-hour ECG records [13]. With a sampling frequency of 360 Hz and an 11-bit resolution within a 10 mV range, each ECG data series consists of two leads, depicted on channels 1 and 2 in Table 1. The first lead is a modified MLII, while the second is one of the modified leads $V_{1}$, $V_{2}$, $V_{4}$, or $V_{5}$ [45]. Some cardiologists independently annotated beat labels, reaching an agreement to resolve discrepancies in diagnoses at the beat and rhythm levels.

The dataset was divided in five classes, in *N*, normal *S*, supraventricular ectopic beat *V*, ventricular ectopic beat *F*, fusion beat between normal and ventricular ectopic beats, and unclassified beat *Q*, in accordance with the Association for the Advancement of Medical Instrumentation (AAMI) guidelines [45,46]. Considering that the samples corresponding to the MIT-BIH dataset represent detailed labels, a mapping established by AAMI is described in Table 2.

According to De Chazal et al. [47], the dataset $DS_{1}$ is used for training and $DS_{2}$ for testing, a standard used in several subsequent studies, as shown in Table 3. The AAMI recommends excluding records that contain pulses from artificial sources, such as pacemaker signals present in records 102, 104, 107, and 217. However, the remaining 44 records, excluding those artificially produced, are generally used in studies that involve the classification of arrhythmias [48].

## 4. Methodology

**Figure 2:**
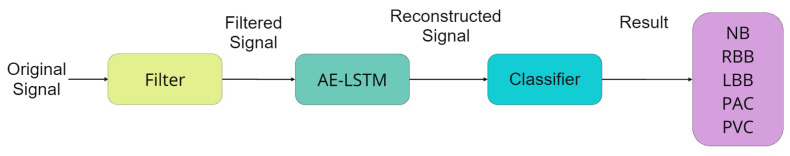
Diagram of the proposed algorithm.

The choice of architecture containing an autoencoder using an LSTM network is based on the characteristics of these techniques to obtain a better result for classifying cardiac arrhythmias. Unlike traditional ML methods in classification tasks, this one does not require manual input of features into the model. In this study, an algorithm will be proposed, as illustrated in Figure 2, formed by a filter for noise treatment, combined with an autoencoder built by an LSTM network, and a classifier also composed of an LSTM network.

### 4.1. Preprocessing

In the preprocessing stage, the signals are treated to reduce interference caused by muscle artifacts; electrode movement; and baseline drift noise in the ECG, also known as drift. Interference manifested as zero-line drift can be caused by poor electrode quality or by the movement of electrostatic objects near input circuits with extremely sensitive bioamplifiers that imply maximum frequencies of ≤1 Hz so as not to impact the final result of the signals to be analyzed [49].

Given that the frequency of an ECG is concentrated between 5 Hz and 20 Hz, the frequency of a muscle movement signal called electromyography ranges from 20 Hz to 5000 Hz, and the frequency of its constituent parts is correlated with the type of muscle, which is typically between 30 Hz and 300 Hz [50]. Consequently, a low-pass filter was chosen to handle the electromyography interference signals. Butterworth, Chebyshev, Bessel, and elliptical filters are the four primary categories of classical filters based on the classification of the filter passband features.

The Butterworth filter is well-known for having a flat passband. To approximate the filter’s system function, the Butterworth function was selected. The squared amplitude function is calculated by

(1) $${\left|H_{a}\left(j\Omega \right)\right|}^{2}=\frac{1}{1+{\left(\frac{\Omega }{\Omega_{c}}\right)}^{2N}},$$

where $\Omega_{c}$ is the frequency at which the amplitude drops to −3 and *N* is the filter’s order, given by

(2) $$N=\frac{1}{2}\frac{lg(\frac{10^{0.1A_{s}}-1}{10^{0.1A_{p}}})}{lg(\frac{\Omega_{s}}{\Omega_{p}})},$$

where $A_{s}$ is the fixed cutoff frequency of the reject band of the low-pass filter, which is 100 Hz. $A_{p}$ is the passband’s cutoff frequency, which is 80 Hz; $\Omega_{s}$ is the reject band’s attenuation, which is 1.6 rad/s; and $\Omega_{p}$ is the pass band’s attenuation, which is 1.4 rad/s [51]. The equation for the filter’s amplitude function can be obtained according to

(3) $$\left|H_{a}\left(j\Omega \right)\right|=\frac{1}{\sqrt{1+{\left(-1\right)}^{N}{\left(\frac{s}{\Omega_{c}}\right)}^{2N}}},s=j\Omega ,\Omega =-\infty ∼+\infty ,$$

where *s* can be solved in Equation (4) in two ways.

(4) $$1+{\left(-1\right)}^{N}{\left(\frac{s}{\Omega_{c}}\right)}^{2N}=0$$

If the value of *N* is even,

(5) $$s=\Omega_{s}e^{j\frac{\left(2k+1\right)}{2N}\pi }.$$

Elseif the value of *N* is odd,

(6) $$s=\Omega_{s}e^{j\frac{k}{2N}\pi }.$$

In the complex frequency domain, the system function’s pole $P_{k}$ is equally spaced on the circle with radius $\Omega_{c}$ Faust et al. [52], and it is given by

(7) $$P_{k}=\left\{\begin{array}{ccc} \Omega_{c}e^{j\frac{2k+1}{2N}\pi }, & N:\mathrm{is}\mathrm{even}, & k=0,1,2\cdots 2N-1\\ \Omega_{c}e^{j\frac{k}{2N}\pi }, & N:\mathrm{is}\mathrm{odd}, & k=0,1,2\cdots 2N-1. \end{array}\right.$$

Only the pole point in the left half-plane of the frequency domain space can be chosen to stabilize the low-pass filter [53]. Following the selection of the stable pole, the low-pass filter’s transfer function can be found as

(8) $$H_{a}\left(s\right)=\prod_{Re[P_{k}]<0}\frac{\Omega_{c}}{s-P_{k}}$$

(9) $$s=\frac{2}{T}\frac{1-z^{-1}}{1+z^{-1}}$$

where *s* is the analog domain transfer function. In this way, the ECG signal from the original lead II is passed through the low-pass filter to reduce electromyography interference.

**Figure 3:**
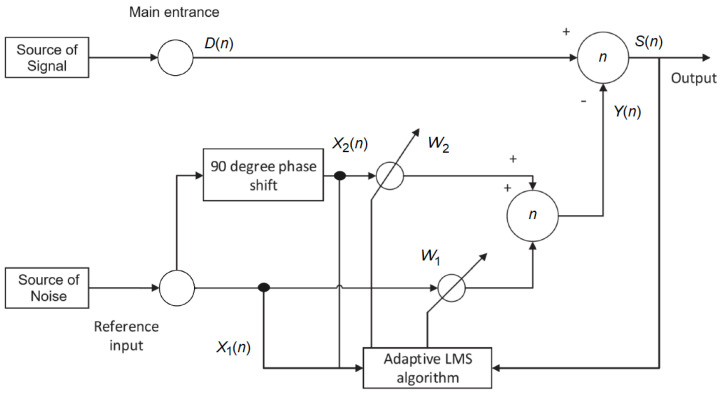
Diagram of the adaptive filter used.

The interference between an electric field and a 50 Hz magnetic field is commonly used to illustrate power frequency interference in electromyography studies. The common filter is impacted if there is a variation. To remove the interference and keep the filter from failing, an adaptive filter is necessary [54]. According to Figure 3, the adaptive filter in this study was designed using the fundamentals of least mean squares (LMS) [55].

The main input channel D(n), as depicted in Figure 3, contains the ECG signal along with power frequency interference noise. Conversely, the reference input includes cross-correlation reference noise. In the same figure, $X_{1}$ and $X_{2}$ denote the power frequency interference and its 90-degree phase-shifted counterpart, respectively, where *n* represents the number of iterations. The component S(n) belongs to the main input channel, while Y(n) denotes the output signal adjusted through the adaptive filtering process. The adaptive filter employed has an order of 2, with weighted coefficients $W_{1}$ and $W_{2}$. These coefficients correspond to the power frequency interference and its 90-degree phase-shifted version, respectively, thereby ensuring effective noise suppression and signal preservation. The equations related to the evaluated adaptive filter are given by:

(10) $$W_{1}\left(n+1\right)=W_{1}\left(n\right)+\mu S\left(n\right)X_{1}\left(n\right),$$

(11) $$W_{2}\left(n+1\right)=W_{2}\left(n\right)+\mu S\left(n\right)X_{2}\left(n\right),$$

(12) $$Y\left(n\right)=X_{1}\left(n\right)W_{1}\left(n\right)+X_{2}\left(n\right)W_{2}\left(n\right),$$

**Figure 4:**
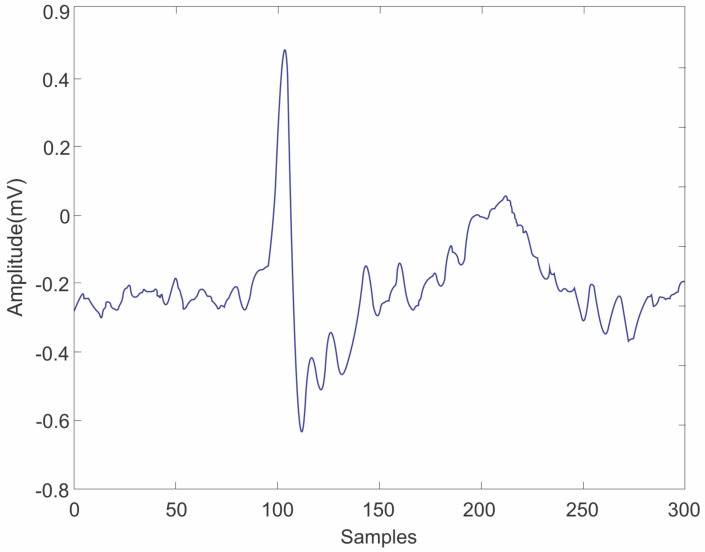
Example of an original signal without passing through the filter.

**Figure 5:**
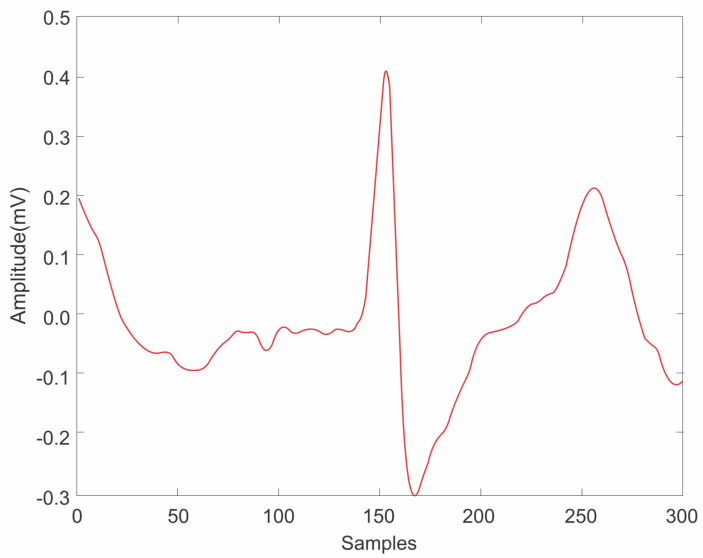
Example of a signal after filtering.

(13) S(n)=D(n)−Y(n),

where μ is the convergence factor set to 0.1. After mitigating the power frequency interference, the weighting coefficient is iteratively adjusted to refine the ECG signal output by repeatedly applying Equations (10) to (13). Upon completion of the various filtering stages, the reconstructed ECG signal waveform is illustrated in Figure 4. It is evident that the noise present in the original signal has been effectively suppressed, as demonstrated by the comparison with the input ECG signal waveform shown in Figure 5.

The continuous beats from the arrhythmia group were utilized for the segmentation of ECG signals. Specifically, the ECG signal was segmented into individual beats, each consisting of 300 samples. The segmentation process was guided by the manually annotated wave peak positions, as recorded in the file *R* provided in the dataset [56]. To preserve all critical information within each heartbeat, the segmentation retained the peak position from *R*, along with 99 samples preceding and 200 samples following the peak. A dataset comprising 97,300 beats was employed in this study, with 75% allocated for training and 25% for testing. The distribution of random samples and the types of arrhythmias considered in this investigation are summarized in Table 4.

### 4.2. Architecture

The combination of an autoencoder and LSTM was selected due to three main benefits. First, LSTM networks are well suited for ECG signals because they easily capture temporal dependencies, modeling long-term relationships between sequential events such as RR intervals and complex QRS durations, which is essential for accurately identifying arrhythmias [57]. Second, the autoencoder acts as a nonlinear filter and implicit regularizer, reducing overfitting and residual noise such as baseline drift that persists after initial preprocessing, thus improving the quality of the extracted features by forcing the model to reconstruct noise-free signals [58]. Finally, training a dedicated autoencoder for each heartbeat class (N, A, L, R, V) allows the model to learn class-specific representations, addressing generalization challenges in imbalanced datasets by mitigating bias towards minority classes such as A, which contains only 3000 samples, while ensuring feature learning tailored to each arrhythmia type.

**Figure 6:**
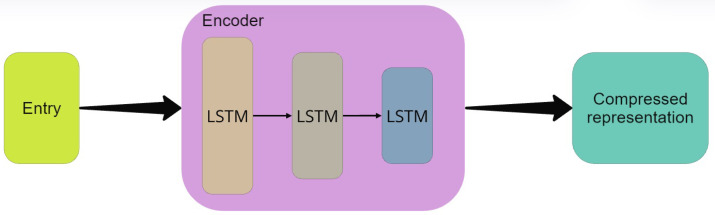
Diagram of the coding layer structure.

As mentioned, to learn the features of the ECG signal, this study proposes an autoencoder based on the LSTM network, whose structure includes an encoding and decoding layer, as well as input and output. The encoding illustrated in Figure 6 is implemented using a three-layer LSTM network. The input to the first layer of the LSTM model is derived from transforming the original ECG signal into a tensor representation. The first LSTM layer comprises 256 units, while the second and third layers are configured with 128 and 64 units, respectively. This hierarchical architecture is designed to progressively extract and encode meaningful temporal features from the input data.

An LSTM unit consists of a cell state $c_{t}$ and three gates that regulate the flow of information. The forget gate $f_{t}$ determines what information from the previous cell state $c_{t-1}$ should be discarded. The input gate $i_{t}$ decides which new information is added to the cell state. Output gate $o_{t}$ controls the output based on the cell state. At each time step *t*, the LSTM performs the following computations:

(14) $$\begin{array}{cc} f_{t} & =\sigma \left(W_{f}x_{t}+U_{f}h_{t-1}+b_{f}\right) \end{array}$$

(15) $$\begin{array}{cc} i_{t} & =\sigma \left(W_{i}x_{t}+U_{i}h_{t-1}+b_{i}\right) \end{array}$$

(16) $$\begin{array}{cc} {\tilde{c}}_{t} & =\tanh\left(W_{c}x_{t}+U_{c}h_{t-1}+b_{c}\right) \end{array}$$

(17) $$\begin{array}{cc} c_{t} & =f_{t}⊙c_{t-1}+i_{t}⊙{\tilde{c}}_{t} \end{array}$$

(18) $$\begin{array}{cc} o_{t} & =\sigma \left(W_{o}x_{t}+U_{o}h_{t-1}+b_{o}\right) \end{array}$$

(19) $$\begin{array}{cc} h_{t} & =o_{t}⊙\tanh\left(c_{t}\right) \end{array}$$

where $x_{t}\in R^{n}$ is the input vector at time *t*, $h_{t}\in R^{m}$ is the hidden state vector, and $c_{t}\in R^{m}$ is the cell state vector. $W_{f},W_{i},W_{c},W_{o}\in R^{m\times n}$ are the weight matrices for input $x_{t}$; $U_{f},U_{i},U_{c},U_{o}\in R^{m\times m}$ are the weight matrices for hidden state $h_{t-1}$; and $b_{f},b_{i},b_{c},b_{o}\in R^{m}$ are the bias vectors. σ represents the sigmoid activation function, tanh is the hyperbolic tangent function, and ⊙ is the element-wise product.

**Figure 7:**
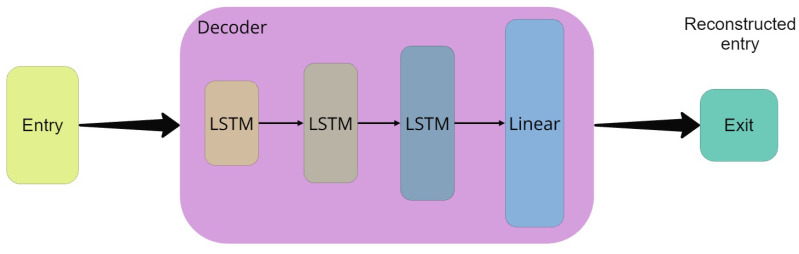
Diagram of the decoding layer structure.

Figure 7 illustrates the construction of the autoencoder’s decoding layer, which comes after the encoding layer and is made up of a linear layer and a three-layer LSTM network. The compressed data from the coding layer are sent to the first layer with 64 units. The network’s second and third layers are configured with 128 and 256 units, respectively. The resulting tensor is then converted by the linear layer into the standard ECG data format (signal reconstruction), which includes the signal properties of the processed data. Details of the parameters used in each layer are shown in Table 5.

Table 6 describes the values applied to the autoencoder LSTM network parameters. After calculating the error, the feedback and backpropagation methods are used to update the model’s internal parameters continuously to reduce the error. We employed Adaptive Moment Estimation (Adam) [59], a stochastic optimization algorithm, with a learning rate of 0.001 to minimize the error. In the future, other optimization techniques may be used. The features of the time series of each kind of beat can be better understood in this way.

Considering the Adam (Adaptive moment estimation) optimizer, let f(θ) denote the objective function to be minimized, where θ represents the parameters of the model. At each time step *t*, the gradient of the objective function to the parameters is computed as $g_{t}=\nabla_{\theta }f_{t}\left(\theta_{t-1}\right)$. The learning rate, denoted by α, controls the step size of the updates. Additionally, $\beta_{1}$ and $\beta_{2}$ are exponential decay rates for the moment estimates, typically chosen such that $\beta_{1},\beta_{2}\in \left[0,1\right)$. The first-moment estimate is denoted by $m_{t}$, and the second-moment estimate is denoted by $v_{t}$.

The Adam optimizer operates by maintaining exponentially decaying averages of past gradients and squared gradients. These averages are used to compute adaptive learning rates for each parameter. The algorithm begins by initializing the first- and second-moment vectors to zero: $m_{0}=0$ and $v_{0}=0$. At each time step *t*, the gradient $g_{t}$ is computed as $g_{t}=\nabla_{\theta }f_{t}\left(\theta_{t-1}\right)$. The first-moment estimate $m_{t}$ is updated using an exponential moving average of the gradient:

(20) $$\begin{array}{c} m_{t}=\beta_{1}m_{t-1}+\left(1-\beta_{1}\right)g_{t}. \end{array}$$

Similarly, the second-moment estimate $v_{t}$ is updated using an exponential moving average of the squared gradient:

(21) $$\begin{array}{c} v_{t}=\beta_{2}v_{t-1}+\left(1-\beta_{2}\right)g_{t}^{2}. \end{array}$$

These estimates are biased towards zero, particularly in the initial time steps, due to their initialization. To address this, bias-corrected versions of the moment estimates are computed. The bias-corrected first-moment estimate is ${\hat{m}}_{t}=m_{t}/(1-\beta_{1}^{t}$), and the bias-corrected second-moment estimate is ${\hat{v}}_{t}=v_{t}/\left(1-\beta_{2}^{t}\right)$. Given these estimates, the parameters are updated using the following rule:

(22) $$\begin{array}{c} \theta_{t}=\theta_{t-1}-\alpha \cdot \frac{{\hat{m}}_{t}}{\sqrt{{\hat{v}}_{t}}+\epsilon }, \end{array}$$

where ϵ is a small constant added to prevent division by zero.

To extract the unique characteristics of every beat, an autoencoder was learned for every class. The classifier uses five layers in the LSTM network. The LSTM network’s first layer consists of 32 units. The output data of the LSTM network were modified by a flattening layer so that they could be interfaced with the subsequent dense layers. A dense layer of 128 units was applied after the flattening layer. During training, a dropout layer was employed at a rate of 0.1 to prevent overfitting. To automatically identify the classifications of beat types from the ECG, a thick layer consisting of five units was positioned as the network’s last layer. Thus, using the autoencoder’s characteristics, the LSTM network categorizes the different types of arrhythmias. Table 7 lists the parameters that were used in the LSTM network for the classification.

The training was divided into two independent stages. First, the autoencoder was trained to reconstruct ECG heartbeats, capturing essential temporal characteristics. In the second stage, the classifier used the latent representations generated by the frozen encoder to learn how to map heartbeats into arrhythmia classes. Both stages employed the Adam optimizer, with early stopping based on validation loss, as shown in Table 6 and Table 7.

### 4.3. Performance Evaluation Metrics

To validate the performance of a model after it has been trained, statistical metrics are used to compare a predicted object against an original object.To evaluate the model’s performance in classification, relevant metrics such as accuracy (Acc), precision (Pr), and sensitivity (Se) were applied [60]. Accuracy is defined as the proportion of correctly predicted instances to the number of predictions made, serving as a fundamental metric for evaluating the performance of classification models. It can be obtained by

(23) $$Acc=\frac{TP+TN}{TP+TN+FP+FN}$$

where *TP* is true positive, *FP* is false positive, *TN* is true negative, and *FN* is false negative.

Precision quantifies the proportion of observations correctly predicted as positive relative to the total number of observations classified as positive by the model. It is commonly expressed as a percentage, as represented in the following equation:

(24) $$Pr=\frac{TP}{TP+FP}$$

With higher precision, the chance of the model classifying a negative observation as positive is lower. Sensitivity, also referred to as recall, is a performance metric that quantifies the proportion of true positive observations correctly identified by the model relative to the total number of truly positive observations. It is a critical measure for evaluating a model’s effectiveness in detecting positive instances, particularly in contexts where minimizing false negatives is essential. The equation is as follows:

(25) $$Se=\frac{TP}{TP+FN}$$

## 5. Results

In the present study, the algorithm was implemented using Python (version 3.13), with a particular emphasis on the TensorFlow-based DL library. The computational hardware employed consisted of an AMD Ryzen 5 3600X six-core processor operating at 3.79 GHz, complemented by 16 GB of random access memory and an 8 GB NVIDIA GeForce RTX 3070 graphics processing unit.

**Figure 8:**
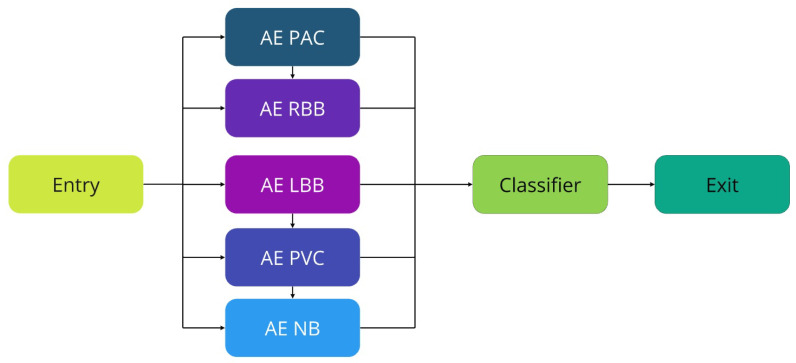
Classification of heartbeat types.

For the experiments, a batch size of 32, a learning rate of 0.001, 100 epochs, and a patience of 5 epochs were considered. The autoencoder was used to be trained on five different types of ECG datasets, as shown in Figure 8. For training, the early stopping option was used when a metric no longer showed improvement. To do this, a patience parameter was set to 5 epochs, meaning that if more than five epochs pass without improvement, training is automatically terminated. For all experiment results, in the model training phase, control parameters were adjusted, as inspired by Yildirim [35], Yildirim et al. [42], and Yildirim et al. [61], including batch size, stations, and the optimizer. The parameters that make up the LSTM network layer were all set to default.

To verify the autoencoder, Mautoencoder was used as a cost function during training as a metric to be monitored. The results for each of the beat types stabilized at 0.1. It was possible to observe that when using a signal different from the one learned, the error increases, stabilizing between 20 and 25. That said, for the classifier to select an output, i.e., reconstructed signal, an allowed error limit between 0.1 and 0.3 was created and included in the inference pipeline.

Considering the data of a heartbeat signal as input for the algorithm (illustrated in Figure 8), five autoencoders, each corresponding to distinct time series features of ECG signals, are utilized. Subsequently, the input ECG signal types are filtered by a threshold based on the reconstruction error of the signals. The heartbeat type is selected as this represents the signal with the most accurate features of the heartbeat type, which will then be used by the classifier.

In Gómez et al. [62], autoencoders with two, three, four, and eight layers of the LSTM network were proposed for encoding and decoding, as shown in Table 8. Five datasets that were not used in training were used to validate the model. The models with five, six, and seven layers presented results very close to those of four and eight layers, in addition to high computational cost. Observing the processing time, the eight-layer model spent on average 29,880 s (8.3 h), while the two-layer model spent on average 12,600 s (3.5 h). It was possible to conclude that for each additional layer, the time interval varies in increments of 3600 s (1 h).

An autoencoder with three layers of LSTM network achieved the best classification result, showing an accuracy of 98.57% in the test; therefore, it was selected for further testing due to its performance. In Table 9, the results for the arrhythmia dataset indicate an overall accuracy of 98.57%, with sensitivity and accuracy ranging from 95.09% to 99.66% and from 96.16% to 97.74%, respectively, for the different arrhythmia classes. The proposed model correctly identifies the vast majority of arrhythmia cases. The classes with the lowest sensitivity were A (95.09%) and *R* (98.08%), which means that the model may have difficulty identifying these arrhythmias in some cases. However, class *N* presented the highest sensitivity (99.34%) and accuracy (99.66%), indicating that the model was effective in identifying this class. Table 10 shows the results for the supraventricular arrhythmia dataset where the overall accuracy achieved by the model was 97.59%. In terms of sensitivity, class A had the lowest rate (89.55%), while class N had the highest rate (99.17%). In terms of accuracy, class V had the lowest rate (92.43%), while class N had the highest rate (98.97%).

The accuracy rate across all classes was consistently high, suggesting that the proposed model effectively classified the majority of the samples with a high degree of correctness. However, this class A showed the lowest sensitivity, indicating that some samples in this class were misclassified. In addition, class V showed the lowest accuracy, indicating that some samples in other classes were misclassified as V. The results presented in Table 10 suggest that the proposed model can correctly classify most of the supraventricular arrhythmia samples. However, there are still some cases where the model may fail, especially in classes A and V.

The MIT-BIH arrhythmia dataset is an important database for cardiac arrhythmia classification research. The arrhythmic ECG signals can be automatically detected using methods such as autoencoders for feature extraction and neural networks for classification. In addition, it provides an automatic and objective method for distinguishing five types of heartbeats from ECG signals.

The amplitudes of various heartbeat types, such as the *P* wave, QRS complex, and *T* wave, exhibit considerable variation across relevant signal bands, making the preprocessing of ECG signals a crucial step in their analysis. However, some heartbeat types display only minimal amplitude differences within the same band, complicating the classification task. Furthermore, the performance of classification models is adversely affected by several factors, including skin polarization effects, issues with the acquisition equipment, and power frequency interference, along with other external noise sources. These factors hinder the accurate differentiation of amplitude variations between certain heartbeat types, leading to degraded classification performance when collecting ECG signals.

An adaptive filter was suggested as a way to improve the model’s performance, and the same classification model was applied without the processing step. The results obtained can be found in Table 11, where it can be seen that there was an increase in false positives for the class V, likely due to high-frequency muscle noise interference and an improvement of 8.57% in accuracy for arrhythmia classification.

Table 12 compares the performance of the full model with simplified variants. Notably, the model exhibited sensitivity to preprocessing quality: signals with uncorrected baseline drift reduced sensitivity by 18%, emphasizing the necessity of robust filtering steps in noisy clinical settings. These findings collectively validate the model’s design choices while delineating actionable improvements for real-world deployment.

While the model achieves high accuracy (98.57%), ablation experiments revealed critical dependencies on architectural and preprocessing components. The removal of the dropout layer caused a 12% increase in overfitting, evidenced by the significant gap between training accuracy (99.1%) and test accuracy (87.3%), which underscores its role in regularization. Similarly, replacing the Adam optimizer with stochastic gradient descent (SGD) doubled the convergence time without meaningful accuracy improvements (98.57% vs. 98.42%), confirming Adam’s superiority for computational efficiency.

The ablation experiments systematically demonstrated the necessity of each architectural component and preprocessing step. As shown in Table 12, removing the adaptive filter degraded overall accuracy and increased false positives due to high-frequency muscle noise. Similarly, removing the autoencoder diminished sensitivity, highlighting its role in extracting physiologically meaningful features such as QRS complex morphology. Notably, the robustness of the preprocessing emerged as a critical factor: uncorrected baseline drift reduced sensitivity, while the absence of the Butterworth filter exacerbated noise-related misclassifications. These findings collectively underscore that the model’s high performance hinges on the integration of the defined architectural components, emphasizing the need to prioritize robust signal preprocessing, especially in noisy environments.

In addition to the MIT-BIH dataset, the model was evaluated on other public datasets to ensure broader applicability. The PTB-XL dataset Wagner et al. [63] comprises 21,801 12-lead ECG recordings and covers a range of cardiac pathologies, including myocardial infarction, ventricular hypertrophy, and bundle branch blocks. This dataset was selected to assess the model’s adaptability to diverse waveform morphologies, such as detecting ST-segment elevation in myocardial infarction. The results considering these datasets (MIT-BIH and PTB-XL) are presented in Table 13.

The proposed model demonstrated robust results on the MIT-BIH dataset, achieving a sensitivity of 97.98%, reflecting its excellent ability to detect true positives even in minority classes such as PAC, and a precision of 97.55%, indicating a low false-positive rate, which is critical for avoiding unnecessary clinical interventions. However, performance varied on other datasets. In PTB-XL, sensitivity dropped to 91.50%, largely due to the complexity of ischemic pathologies such as myocardial infarction, which require detailed morphological analysis of subtle waveform changes (e.g., ST-segment deviations). Precision also dropped to 89.30%, attributed to the high variability of T-wave patterns across infarct subtypes.

While performance on the PTB-XL dataset reflects challenges inherent in complex ischemic pathologies (e.g., T-wave variability and ST-segment deviations), the lower performance on PTB-XL suggests that the current architecture, while optimized for arrhythmias, presents potential model adaptability to diverse waveform morphologies, highlighting its potential for broader application. Different models have been proposed in the literature to automatically detect arrhythmia ECG signals, with some focusing on ECG data classification from MIT-BIH arrhythmia data. Table 14 provides a summary of models documented in the literature, all evaluated on the same dataset. For this comparison, the results obtained from the validation set were utilized.

For example, Sahoo et al. [64] proposed an approach that utilizes discrete wavelet transform-based multiresolution techniques to extract features for the classification and complete detection of heartbeats. Li et al. [65] examined the application of independent component analysis (ICA), principal component analysis (PCA), and discrete wavelet transform (DWT) to extract features from ECG signals in both the time and frequency domains, respectively, aiming for comprehensive classification through support vector machines (SVM). Yeh et al. [66] introduced linear discriminant analysis (LDA) for directly extracting morphological features from ECG components, subsequently utilizing these features for heartbeat classification, and Elhaj et al. [67] also achieved promising results using ICA and higher-order spectra (HOS) by combining ANNs and SVM. Furthermore, Martis et al. [68] performed beat segmentation in ECG signals, followed by the application of PCA, ultimately achieving accurate classification through SVM with a radial basis function (RBF) kernel.

**Table 14:** State of the art in automatic arrhythmia detection.

Author	No. de Beats	Models	Acc (%)	Se (%)	Pr (%)
[66]	102,060	LDA	96.23	92.49	
[68]	1247	SVM-RBF	98.48	98.90	
[65]	1800	DWT, SVM, kernel ICA, PCA	98.8	98.50	98.91
[67]	110,094	DWT, PCA, HOS, ICA, SVM-RBF	98.91	98.91	
[69]	100,389	CNN	92.70		
[70]	83,648	CNN	99.00	93.90	
[64]	109,494	DWT, SVM	98.39	99.87	99.21
[71]	109,449	CNN	94.03	96.71	
[4]	21,709	CNN	92.50	98.09	
[72]	16,499	LSTM, CNN	98.10	97.50	98.69
[35]	7376	LSTM	99.39		
Proposed	97,300	Autoencoder-LSTM	98.57	97.98	97.55

Several DL models have also been proposed for arrhythmia detection and classification: the CNN was used by Acharya et al. [71], Oh et al. [72] presented a hybrid approach using CNN and LSTM networks for arrhythmia detection, and Acharya et al. [4] presented another way to detect and classify ECG signals. Other studies using DL models have also obtained satisfactory results close to the state of the art, such as in [69,70] and Yildirim [35].

The proposed model outperformed pure CNNs in accuracy, as it captures long-term temporal dependencies that CNNs ignore. In environments with electromyography (EMG) interference, our model achieved a better accuracy than the CNNs presented by Acharya et al. [4,71] and Zubair et al. [69], as shown in Table 14 on the same noisy dataset. This is because the autoencoder acts as a nonlinear adaptive filter, suppressing high-frequency artifacts that degrade the performance of spatial convolution-based models. Compared to traditional methods of Martis et al. [68] and Li et al. [65], the difference highlights the advantage of automatic feature learning without manual intervention. The hybrid CNN–LSTM approach, proposed by Oh et al. [72], obtained close results to ours, suggesting that the integration of spatial and temporal features may be promising. Our results are in line with Yildirim [35], who reported 99.39% accuracy with pure LSTMs but on smaller datasets. In contrast, CNNs such as that of Kiranyaz et al. [70] achieve up to 99% accuracy but require complex augmentation adjustments for generalization.

Table 15 describes the results of recent studies that used different models to detect arrhythmias in ECGs in the MIT-BIH dataset. Gómez et al. [62] presented an ECG classification model using a Bi-LSTM network inspired by Yildirim et al. [61], which involves a five-layer LSTM network, achieving an accuracy of 82.10%. Tang and Tang [73] used an LSTM network in addition to extracting morphological features from the ECG signal, achieving an accuracy of 82.14%. Hammad et al. [74] used a deep neural network combined with a genetic algorithm to extract features and classify ECG signals. Chen et al. [75] utilized a hybrid arrhythmia detection model with CNN and Bi-LSTM. Wang et al. [76] used CNNs to implement methods for multi-scale feature extraction and the complementary integration of cross-scale information in the analysis of ECG signals.

The model proposed in this paper demonstrates better performance than the state-of-the-art models in multiclass arrhythmia classification, achieving 98.57% accuracy across five distinct classes, surpassing the 98.00% accuracy reported by Hammad et al. [74] using a DNN architecture while also delivering a 1.75% improvement in precision (97.5% vs. 95.80%), indicative of reduced false-positive rates critical for clinical triage. Notably, the model maintains a balanced sensitivity–precision profile (97.98% Se vs. 97.55% Pr), contrasting with sensitivity-prioritized approaches like the DNN in [74] (99.70% Se at the expense of precision). This equilibrium is particularly advantageous in clinical settings where false positives incur operational costs, such as unnecessary patient alarms or resource allocation.

The presented architecture outperformed hybrid CNN + Bi-LSTM designs such as the one presented by Chen et al. [75], which exhibit markedly lower sensitivity (74.89%) for four-class tasks, likely due to poor rare-class detection (e.g., fusion beats). By integrating an LSTM-based autoencoder, the proposed system enhances generalization capabilities even under dataset imbalance, as evidenced by its robust V-class sensitivity. Furthermore, our model outperformed pure LSTM implementations, like those presented by Gómez et al. [62] and Tang and Tang [73], which plateau at <= 82% accuracy for binary classification, by leveraging hierarchical temporal feature extraction. This enables the model to capture subtle electrophysiological patterns (e.g., QRS widening in V-type beats) while maintaining computational efficiency at a five-class accuracy of 16.57% higher than two-class LSTM benchmarks. Note that, compared to the previous models, the model proposed in this study has a superior result.

This study used an adaptive filter and performed feature extraction and classification of ECG data using deep learning methodologies. Although there are other studies using LSTM networks to classify ECG signals [35,62,72,73], the main difference is the use of an autoencoder to extract features from ECG signals, which improved the classification performance of the LSTM network, differing from the approaches described in Table 15. Although the classification accuracy is not superior to that reported in [35], a new classification tool is presented along with the construction of a new format to extract signal features instead of using ECG signals directly.

## 6. Discussion

In this study, an algorithm was proposed that uses an autoencoder for feature extraction and a classifier using the LSTM network, in which it was possible to find a satisfactory result in classification, especially in multiclass task scenarios, as described in the two previous tables. The balance between sensitivity (97.98%) and precision (97.55%) positions the model as a promising tool for automated screening in telemedicine, reducing cardiologists’ workload. The proposed model achieved 98.57% accuracy in the classification of five classes, surpassing even recent approaches as mentioned earlier, which reported 98.0% for the same number of classes. This difference can be attributed to the preprocessing stage of the ECG signal since the treatment of the signals sought to reduce electromyography interference, electrode movement, and baseline fluctuation noise in the ECG. To improve the model’s performance, an adaptive filter was proposed in addition to signal segmentation, with which it was possible to identify that noise affects the extraction of signal characteristics and, consequently, their classification and the integration of the autoencoder, by acting as an implicit regularization mechanism, filtering out residual noise and learning robust representations even in imbalanced classes (e.g., class A with only 3000 samples).

To process and extract features from the signal, an autoencoder was proposed, trained on five classes of ECG datasets. The error between output and input in the autoencoder was found to be stable at a value below one. Using the performance metrics, it was possible to identify the ideal size of the autoencoder and adjust the hyperparameters, in which the three-layer model obtained the best performance. It was also possible to identify the satisfactory performance of the model classification tasks applied to ECG signal banks. Compared to others, it obtained greater accuracy, although the structure is simpler in terms of architecture size, containing a small variation in the hyperparameters to provide easy reproduction and also to mitigate the chances of problems such as gradient disappearance.

The proposed model for ECG signal classification uses an ECG time series judgment that uses a threshold containing a rule to select the smallest error as input for classification. By thoroughly taking into account the useful judgment elements suggested in earlier research, the model’s output will be more trustworthy. However, the proposed objectives were achieved and the results obtained were satisfactory.

Moreover, while models such as Chen et al. [75] suffer from reduced sensitivity due to the difficulty in distinguishing subtle patterns (e.g., fusion beats F vs. ventricular V), our approach maintains 97.98% sensitivity due to the ability of LSTMs to model long-term temporal dependencies. The high accuracy (97.55%) of the model, in contrast to DNN, suggests a lower rate of false positives, an essential criterion for clinical applications where misdiagnoses can lead to unnecessary interventions. For example, a false positive in ventricular (V) beats could result in inappropriate antiarrhythmic medication. The autoencoder contributes to this balance by reducing overfitting and prioritizing physiological features over noise artifacts. This advantage is particularly relevant when compared to pure LSTMs, which, although efficient for two classes, show limited accuracy in more complex problems.

Approaches using a CNN + Bi-LSTM architecture achieved 96.77% accuracy but with critically low sensitivity, indicating difficulty in detecting minority classes. This problem is mitigated in our model by two-stage training: in the first phase, the autoencoder learns generalizable representations; in the second, the classifier focuses on discriminative patterns, avoiding bias towards majority classes. Despite the advancements, several limitations must be acknowledged. First, the model’s performance is heavily reliant on adaptive preprocessing: without the adaptive filter, accuracy drops significantly to 90.83% (Table 11), indicating sensitivity to high-frequency noise. Second, the computational cost of the two-stage training process is a challenge for deployment on low-power wearable devices.

This work demonstrates that combining autoencoders with LSTMs provides unique advantages for ECG classification, outperforming conventional approaches in multiclass scenarios. Despite its limitations, the proposed architecture sets a new benchmark for automated cardiac diagnostic systems by balancing high accuracy, efficiency, and adaptability.

## 7. Conclusions

The growth of automation techniques for the recognition and classification of ECG signals highlights the importance of resources to help healthcare professionals prevent and diagnose alterations in heartbeat patterns, such as arrhythmias, whose traditional detection methods are time-consuming, error-prone, and subjective, making early diagnosis crucial to reduce the risk of cardiovascular events. The proposed algorithm, selected after a literature search, uses an autoencoder with LSTM layers to extract features from ECG signals, trained in five types of ECG data with an important preprocessing step to reduce interference and noise. An adaptive filter and signal segmentation were proposed to improve the model’s performance, with the three-layer autoencoder achieving the best performance in classification tasks. The results showed that it is not necessary to manually define or adjust the model’s input parameters, presenting satisfactory results compared to other approaches.

Further investigations could compare the LSTM-based autoencoder’s performance with designs like transformers. Furthermore, it is necessary to generate explainable artificial intelligence to provide healthcare practitioners with insights into model predictions by clarifying the underlying decision-making processes.

## Figures and Tables

**Table 1:** Types of leads and grouping in the MIT-BIH dataset.

Channel 1	MLII	MLII	MLII	$V_{5}$	$V_{5}$	MLII
Channel 2	$V_{2}$	$V_{4}$	$V_{5}$	$V_{2}$	MLII	$V_{1}$
Patient IDs	103 117	124	100 123	102 104	114	101 105 106 107 108 109 111 112 113 115 116 118 119 121 122 200 201 202 203 205 207 208 209 210 212 213 214 215 217 219 220 221 222 223 228 230 231 232 233 234

**Table 2:** Mapping of MIT-BIH to AAMI classes.

AAMI	MIT-BIH	Description
*N*	N L R e j	Normal beat Left bundle branch block Right bundle branch block Atrial escape beat Nodal (junctional) escape beat
*S*	A a J S	Premature atrial contraction Aberrant premature atrial contraction Premature nodal (junctional) beat Premature supraventricular contraction
*V*	V E	Premature ventricular contraction Ventricular escape beat
*F*	F	Fusion of normal and ventricular beats
*Q*	P f U	Paced heartbeats Fusion of normal and paced beats Unclassified beats

**Table 3:** Organization of the Patient IDs dataset according to AAMI.

${\mathrm{DS}}_{1}$-Training	${\mathrm{DS}}_{2}$-Test
101, 106, 108, 109, 112, 114, 115, 116, 118, 119, 122, 124, 201, 203, 205, 207, 208, 209, 215, 220, 223, e 230	100, 103, 105, 111, 113, 117, 121, 123, 200, 202, 210, 212, 213, 214, 219, 221, 222, 228, 231, 232, 233, e 234

**Table 4:** Types of arrhythmias and data distribution.

Types of Arrhythmias	Amount (Beats)
Premature atrial contraction	3000
Left bundle branch block	8000
Right bundle branch block	7200
Premature ventricular contraction	7100
Normal beat	72,000
Total beats	97,300

**Table 5:** Details and parameters of each autoencoder layer.

Layer	Unit Size	Parameters
LSTM	256	* RS = T
LSTM	128	* RS = T
LSTM	64	* RS = T
LSTM	64	* RS = T
LSTM	128	* RS = T
LSTM	256	* RS = T
Linear	300	

* Return Sequences (RS) = True (T).

**Table 6:** Autoencoder LSTM network parameters.

Parameters	Values
activation function	tanh
recurrent activation function	sigmoid
use bias	True
kernel initializer	glorot uniform
recurrent initializer	orthogonal
bias initializer	zeros
unit forget bias	True
epochs	100
lot size	32
patience	5
learning rate	0.001

**Table 7:** Parameters used for LSTM network classification model.

Layer	Unit Size	Parameters
LSTM	32	Return Sequences = True
Flat	-	-
Dense	128	activation function = ReLU (Rectified Linear Unit)
Dropout	-	rate = 0.1
Dense	5	activation function = softmax

**Table 8:** Accuracy using an autoencoder with an LSTM network versus different numbers of layers.

Layers	2	3	4	8
Training (%)	93.77	98.31	96.64	98.28
Testing (%)	92.47	98.57	97.11	97.13

**Table 9:** Confusion matrix applied to the MIT-BIH arrhythmia dataset.

		Predicted					Acc (%)	Se (%)	Pr (%)
		A	L	N	R	V			
Original	A	252	1	8	0	1	98.57	95.09	96.16
	L	5	677	14	4	0		98.54	96.71
	N	5	5	6279	6	5		99.34	99.66
	R	2	3	10	615	1		98.08	97.46
	V	1	1	10	2	607		98.85	97.74

**Table 10:** Confusion matrix applied to the MIT-BIH supraventricular arrhythmia dataset.

		Predicted					Acc (%)	Se (%)	Pr (%)
		A	L	N	R	V			
Original	A	240	5	5	8	4	97.59	89.55	91.60
	L	10	647	20	13	10		94.87	92.43
	N	7	18	6235	20	20		99.17	98.97
	R	5	4	4	613	5		92.32	97.15
	V	6	8	23	10	574		93.64	92.43

**Table 11:** Confusion matrix applied to the MIT-BIH arrhythmia dataset without preprocessing.

		Predicted					Acc (%)	Se (%)	Pr (%)
		A	L	N	R	V			
Original	A	171	26	28	23	14	90.83	61.96	65.27
	L	32	517	98	32	21		75.36	73.86
	N	58	121	5932	129	60		96.75	94.16
	R	7	8	20	581	15		74.58	92.02
	V	8	14	53	14	532		82.87	85.67

**Table 12:** Model performance in different configurations.

Configuration	Acc (%)	Se (%)	Pr (%)
Full Model	98.57	97.98	97.55
Without Adaptive Filter	90.83	61.96	65.27
Without Autoencoder	86.40	72.10	68.90
1 LSTM Layer (Encoder)	92.47	89.20	88.50
Dropout Removed	87.30	85.10	84.80

**Table 13:** Revised comparative analysis of ECG datasets.

Dataset	Samples	Classes	Acc (%)	Se (%)	Pr (%)
MIT-BIH (Considered)	97,300	5	98.57	97.98	97.55
PTB-XL	21,801	20	93.20	91.50	89.30

**Table 15:** Recent studies on the automatic detection of arrhythmias from MIT-BIH dataset.

Author	Models	No. of Classes	Acc (%)	Se (%)	Pr (%)
[62]	Bi-LSTM, LSTM	2	82.14	87.14	79.22
[73]	LSTM	2	82.10	78.40	84.79
[75]	CNN, Bi-LSTM	4	96.77	74.89	81.24
[74]	DNN	5	98.00	99.70	95.80
Proposed	Autoencoder-LSTM	5	98.57	97.98	97.55

## Data Availability

Dataset available on request from the authors.
